# Inspiratory muscle training in the healthy adult: The relationship between load, perception, and oxygen consumption

**DOI:** 10.1111/cpf.70047

**Published:** 2026-01-21

**Authors:** Timothy O. Jenkins, Dan Stieper Karbing, Stephen Edward Rees, William Scott, Christos Aristidou, Mathias Krogh Poulsen, Michael I. Polkey, Vicky MacBean

**Affiliations:** ^1^ Rehabilitation and Therapies Department, Royal Brompton and Harefield Hospitals, Heart, Lung and Critical Care Group Guy's and St Thomas' NHS Foundation Trust London UK; ^2^ College of Health, Medicine and Life Sciences, Department of Health Sciences Brunel University of London UK; ^3^ Respiratory and Critical Care Group, Department of Health Science and Technology Aalborg University Aalborg Denmark; ^4^ Intramedic A/S Egebækvej 98d Nærum Denmark; ^5^ Department of Respiratory Medicine, Heart, Lung and Critical Care Group Guy's and St Thomas' NHS Foundation Trust London UK

**Keywords:** critical care, exercise, oxygen consumption, resistive breathing, respiration pattern, respiratory muscle

## Abstract

**Background:**

Inspiratory muscle training (IMT) is used in a broad range of populations to improve the strength and endurance of the respiratory muscles, to improve both athletic performance and clinical outcomes. However, the optimal approach to IMT remains uncertain, and IMT is frequently declined in the clinical setting. This study aimed to measure oxygen consumption (VO_2_) and perceived difficulty and unpleasantness during commonly cited IMT loads.

**Methods:**

Thirty participants performed IMT at 4cmH_2_O and 30%, 50% and 80% of their maximal inspiratory strength (PImax). VO_2_ was measured using indirect calorimetry. After each load, a visual analogue scale was used to rate breathing difficulty (VAS‐D) and unpleasantness (VAS‐U)

**Results:**

Median (IQR) VO_2_ was 4.42 (3.36–4.82) mL/min/kg at baseline, increasing to 4.90 (4.11–5.03) mL/min/kg, 4.38 (3.69–5.23) mL/min/kg, 4.64 (4.09–5.28) mL/min/kg and (4.82–6.51) mL/min/kg after IMT at 4cmH_2_O and 30, 50 and 80% PImax respectively (Friedman's ANOVA *p* < 0.001). VO_2_ increased by 0.013 mL/kg/min for every 1% of PImax increase in IMT load. Perceived difficulty and unpleasantness increased with IMT load. PImax significantly influenced the load‐perception relationship: slope (95% CI) of load versus VAS‐D in the combined model 0.37 (0.09–0.65)mm/%PImax, *p* = 0.01), additional influence of baseline PImax 0.003 (0.001–0.005) mm/%PImax/cmH_2_O, *p* = 0.009.

**Conclusions:**

IMT causes a load‐dependent increase in VO_2_, with marked increases in breathing difficulty and unpleasantness at higher loads. The additional impact of the absolute magnitude of load provides insight into the perception of respiratory effort. These data help understand the factors that influence IMT prescription, in terms of exercise response and acceptability.

## 1 INTRODUCTION

Inspiratory muscle training (IMT) is an intervention used in a broad range of populations, including performance sport (Xavier et al., 2025), and in pathologies where diaphragm weakness exists (Levine et al., 2008), or, for example, where the diaphragm is at a mechanical disadvantage (Ottenheijm et al., 2008). IMT aims to improve the strength and endurance of the diaphragm and extra‐diaphragmatic muscles through the application of controlled resistance during inspiration (Beaumont et al., 2018; Bissett et al., 2016; Charususin et al., 2018a; Schultz et al., 2018; Vorona et al., 2018).

In athletes, IMT can increase inspiratory strength, pulmonary function, and physical performance (Xavier et al., 2025). It is frequently used as a supplementary tool to improve exercise performance, and is thought to reduce perceived breathlessness and attenuation of peripheral muscle fatigue, the main limitations of physical exercise (Martins de Abreu et al., 2019; Eastwood et al., 2001; HajGhanbari et al., 2013; Xavier et al., 2025). In clinical populations, IMT can improve inspiratory strength, yet improvements in clinically important outcomes vary; In heart failure, IMT can improve quality of life and exercise capacity (using the 6‐min walk test) (Siddiqi et al., 2025), whereas in chronic obstructive pulmonary disease (COPD), three large randomized controlled trials found improvements in inspiratory strength, but these improvements failed to translate into improvements in clinically important outcomes such as quality of life, dyspnoea or exercise capacity (Beaumont et al., 2018; Charususin et al., 2018a; Schultz et al., 2018). In critical care, IMT is commonly used to ameliorate the effects of ventilator induced diaphragm dysfunction in mechanically ventilated patients, where it can improve maximal inspiratory pressure (Elkins & Dentice, 2015; Hearn et al., 2022; Vorona et al., 2018). However, there is conflicting data surrounding improvements in clinically important outcome measures such as likelihood of weaning success or intensive care length of stay, partly owing to differing training regimes and methodological heterogeneity of the current literature (Van Hollebeke et al., 2025; Vorona et al., 2018).

Despite a plethora of trials investigating the effects of IMT, the optimal approach to IMT remains uncertain (Shei et al., 2022; Vorona et al., 2018). Training regimes in athletes vary widely across the literature with resistance levels set anywhere between 20% and 90% of the persons maximal inspiratory strength (PImax) or, in clinical populations, resistance set initially between 20% and 70% Pimax and increased to the maximum tolerable by the patient; commonly around 80% of their PImax (Beaumont et al., 2018; Charususin et al., 2018a; Elkins & Dentice, 2015; Martin et al., 2011; Schultz et al., 2018). There is a clear need for optimization of the IMT stimulus (Ballesteros‐Reviriego et al., 2024; Poddighe et al., 2025; Shei et al., 2022; Van Hollebeke et al., 2022); additionally, exploring perceived difficulty and unpleasantness of IMT may provide insight into the experience and tolerance of IMT, which may be of use to clinicians to help improve adherence of the intervention, which can be as low as 30% in clinical populations (Bissett et al., 2023).

Quantification of oxygen consumption (VO_2_) during IMT may provide guidance on determining the appropriate IMT training dose for healthy and clinical populations, to determine if commonly cited IMT loads translate to an increase in VO_2_, to achieve a training effect on the respiratory muscles. Our work in mechanically ventilated patients (Jenkins et al., 2023) found IMT caused a significant and load‐dependent increase in VO_2_, similar to results found in healthy subjects (Eastwood et al., 1998); however, these healthy data used set inspiratory resistances, not proportionate to the participant's maximal inspiratory pressure (PImax) (Eastwood et al., 1998). In mechanically ventilated patients, VO_2_ is modulated by baseline PImax, indicating that individualized IMT prescription may be of value (Jenkins et al., 2023).

The aim of this study was to measure the response of the healthy respiratory system to commonly prescribed IMT loads. Concurrently, we measured perceived difficulty and unpleasantness of IMT whilst examining the influence of inspiratory muscle strength on respiratory load perception.

## 2 MATERIALS AND METHODS

### 2.1 Participants

Participants were included if they were aged 18 or over. Exclusion criteria were lung or heart disease requiring medication, any disorder of the eardrum (such as a ruptured eardrum), or muscular/neurological condition affecting the chest and/or torso. The study was conducted according to the Declaration of Helsinki and approved by the College of Health, Medicine and Life Sciences Research Ethics Committee at Brunel University London. All participants provided written informed consent. Between 19 July and 13 October 2022, 30 participants were recruited.

## 3 EXPERIMENTAL DESIGN

### 3.1 PImax and pressure measurements

PImax was measured with a differential pressure transducer (MK1S pressure transducer, GM Instruments, Irvine, Scotland) during a sustained maximal inspiratory effort against an occlusion from FRC, and calculated as the greatest 1‐second mean pressure. Measurement was repeated until three measures within 10% of one another were obtained (Laveneziana et al., 2019), with the highest value reported. Pressure at the airway opening was measured continuously during the test period with the MK1S transducer, attached via a port in the bacterio‐viral filter attached between the facemask and the IMT device. The pressure signal was digitized (PowerLab 4/35, ADInstruments) and displayed on LabChart7 software (ADInstruments). Inspiratory time and respiratory cycle time were analyzed offline using LabChart7.

## 4 PROTOCOL

### 4.1 Metabolic measurements

Oxygen consumption and carbon dioxide production were measured using breath‐by‐breath indirect calorimetry (Beacon Caresystem, Mermaid Care A/S, Norresundby, Denmark). The Beacon device has shown equivalence to other widely‐used devices, including the E‐sCOVX® (GE Healthcare, Helsinki, Finland) (Poulsen et al., 2019) and the QUARK RMR (COSMED, Rome, Italy) (Slingerland‐Boot et al., 2022). The Beacon Caresystem has clinically relevant advantages compared to other methods of oxygen consumption measurement (such as a Douglas bag), including simple connection to the participant and no need for calibration. The system also gives an indication of real‐time VO_2_. The respiratory gas flow sensor (SPIRT^TM^ flow sensor, Adult, Artema Technology, Germany) from the Beacon Caresystem was connected to the participant's mask throughout the study procedure. Baseline VO_2_ was measured as a 2‐min average during a steady state of relaxed breathing.

**FIGURE 1:**
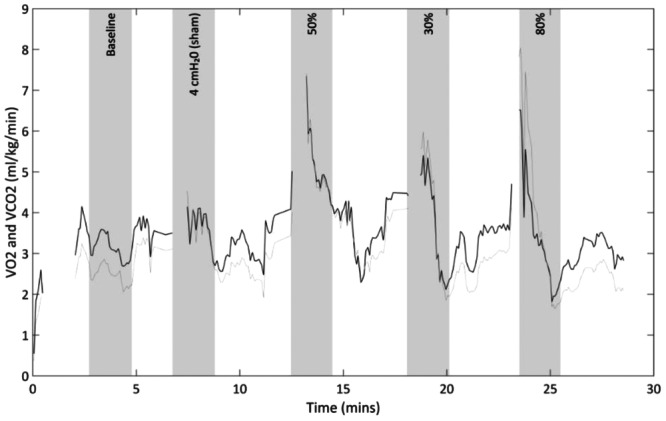
Title: A typical participant's VO_2_/VCO_2_ data. Legend: Black line shows VO_2_ signal, grey line shows VCO_2_ signal. Shaded grey area shows 2‐min average taken at baseline and after each training load.

We used a 2‐min post‐load average to reflect the oxygen deficit incurred by respiratory exercise (Buitrago et al., 2014; Jenkins et al., 2023; McCool et al., 1989). The Beacon system is unable to measure VO_2_ during IMT as the VO_2_ signal is distorted by the substantial negative pressures generated during IMT. Figure 1 shows a typical full protocol of IMT with the VO_2_ and VCO_2_ signal output from the Beacon Caresystem.

## 5 IMT

**FIGURE 2:**
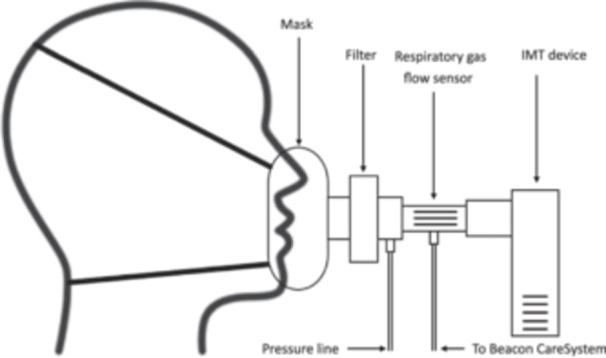
Title: Experimental setup.

Inspiratory threshold loading was applied at 30%, 50% and 80% of each participant's PImax using an inspiratory muscle training device. To enable the most accurate load to be applied, IMT was applied using a POWERbreathe Plus IMT (light, medium or heavy resistance) or a POWERbreathe Medic Plus IMT). A “sham” load at 4cmH_2_O was delivered using an inverted Philips Threshold PEP device as previously described (Martin et al., 2011). The order of load was randomized using Sealed Envelope^TM^ (Oxford, UK), and participants were blinded to the applied IMT load. The participant was instructed to initiate breaths from functional residual capacity and encouraged by the investigator to inhale deeply against the load for 12 breaths at their own pace. No set breathing frequency or pattern was imposed, as this would have differential effects between participants on perception of load, and not reflect IMT in real‐world practice. After each load, participants performed 5 min of tidal breathing to allow for VO_2_ measurement and recovery. The experimental setup is shown in Figure 2.

### 5.1 Measurement of breathing difficulty and unpleasantness

A 100 mm visual analogue scale was used to rate breathing difficulty (VAS‐D) and breathing unpleasantness (VAS‐U) immediately after each load. Anchors were ‘no breathing difficulty at all’ and ‘breathing feels as difficult as I could possibly imagine’, and ‘not unpleasant at all’ and ‘breathing feels as unpleasant as I could possibly imagine’, respectively.

### 5.2 Raw data analysis

Tension‐time index of the respiratory muscles (TTmus) was calculated as mean airway pressure divided by PImax, multiplied by the respiratory duty cycle (Ramonatxo et al., 1995). VO_2_ for each IMT load was calculated using MatLab (R2022a, MathWorks, Massachusetts, USA), using event markers inputted on the Beacon Caresystem during the study procedure.

### 5.3 Statistical analysis

Data were assessed for normality using a histogram and Shapiro–Wilk test. Baseline characteristics were reported using descriptive statistics and presented as median (IQR). Friedman's ANOVA was used to examine whether VO_2_, VAS‐D, and VAS‐U differed with IMT doses. Dunn's post hoc testing (using Bonferroni correction for multiple comparisons) was used to explore differences in VO_2_, VAS‐D, and VAS‐U at each IMT load. Difference in VO_2_, VAS‐D, and VAS‐U between females and males at each IMT load was compared using a Mann–Whitney test. Linear mixed effects modelling (LMM) was used to quantify the relationship between IMT load and VAS‐U/VAS‐D, and the influence of PImax. LMM was used to quantify the relationship between IMT load and VO_2_, between TTmus and VO_2_, and between respiratory duty cycle (Ti/Ttot) and IMT dose and VO_2_. Analysis of all data were conducted by persons blinded to the intervention loads. A two‐tailed level of *p* < 0.05 was considered statistically significant. Statistical analyses were performed by SPSS Version 29.0.1.0 for Windows (IBM, Inc.).

## 6 RESULTS

Baseline characteristics are presented in Table 1.

**TABLE 1:** Baseline participant characteristics.

Variable	*n* = 30
Sex (Female: Male)	18: 12
Age (years)	32.0 (24.3–44.5)
Height (cm)	173 (163–179)
Weight (kg)	73.3 (64.5–84.6)
PImax (cmH_2_O)	119 (48)
PImax (%predicted)	101.1 (37.7)

*Note*: Data presented as median (IQR) or mean (SD) unless otherwise stated. PImax: maximum inspiratory pressure.

### 6.1 Influence of load on VO_2_

**FIGURE 3:**
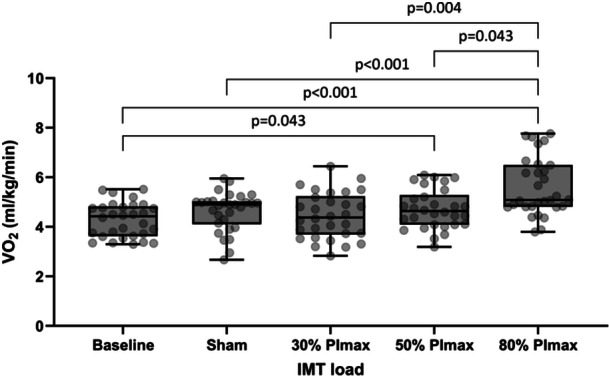
Title: Box and whisker plot detailing oxygen consumption (mL/min/kg) at baseline, 4cmH_2_O (sham) and at 30, 50 and 80% IMT dose. *p*‐values shown for significant relationships only. Individual data points are shown in background.

VO_2_ differed significantly with IMT load (*p* < 0.001) (Table 2 and Figure 3). *Post hoc* comparisons revealed significant differences in VO_2_ between baseline and 50% and 80% IMT doses (*p* = 0.043 and *p* < 0.001 respectively), sham and 80% (*p* < 0.001), 30% and 80% (*p* = 0.004), and 50% and 80% (*p* = 0.043). There was no significant difference in VO_2_ between females and males at all IMT loads.

**TABLE 2:** Influence of IMT load on VO_2_, VAS‐D, and VAS‐U.

	Baseline	Sham (4cmH_2_O)	30% PImax	50% PImax	80% PImax	*p* value (Friedman's ANOVA)
VO_2_ (mL/min/kg)	4.42 (3.36–4.82)	4.90 (4.11–5.03)	4.38 (3.69–5.23)	4.64 (4.09–5.28)	5.10 (4.82–6.51)	<0.001
VAS‐D (mm)	5 (0–10)	11 (3–18)	29 (13–52)	41 (29–64)	73 (48–93)	<0.001
VAS‐U (mm)	6 (3–17)	9 (3–12)	26 (8–55)	42 (20–55)	79 (50–94)	<0.001

*Note*: Data presented as median (IQR). VO_2_: oxygen consumption; VAS‐U: breathing unpleasantness visual analogue scale; VAS‐D: breathing difficulty visual analogue scale.

LMM showed a significant dose‐response relationship between IMT load and VO_2_ Intercept (95% confidence interval) 4.30 (4.03–4.57) mL/kg/min (*p* < 0.001). Slope (95% CI): 0.013 (0.009–0.018) mL/min/kg per %PImax (*p* < 0.001); indicating VO_2_ increases by 0.013 mL/kg/min for every 1% of PImax increase in load.

VO_2_ was also significantly related to TTmus. Intercept (95% CI) 4.28 (3.98–4.58) mL/min/kg, *p* < 0.001. Slope (95% CI) 3.74 (2.67–4.81) mL/min/kg per unit increase in TTmus (*p* < 0.001).

Respiratory duty cycle did not have a significant additional influence on the relationship between IMT dose and V̇O_2_ (*p* = 0.294), suggesting breathing pattern or rate had no impact on metabolic oxygen consumption.

### 6.2 Influence of load on VAS‐D and VAS‐U

**FIGURE 4:**
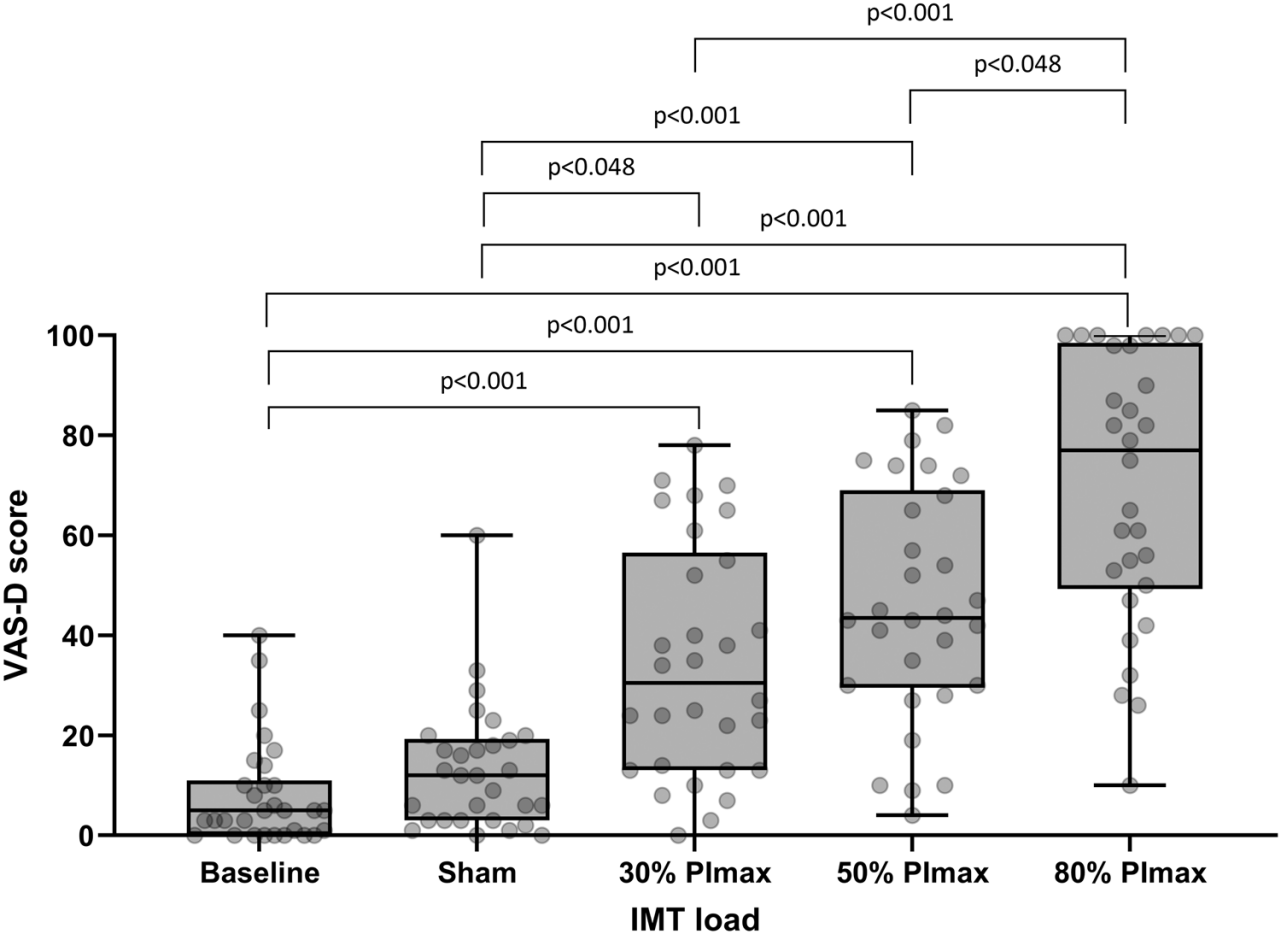
Title: Box and whisker plots detailing changes in visual analogue scale values for breathing difficulty (VAS‐D) at 0 (baseline), 4cmH_2_O (sham) and at 30, 50 and 80% IMT load. *p*‐values shown for significant relationships only. Individual data points are shown in background.

**FIGURE 5:**
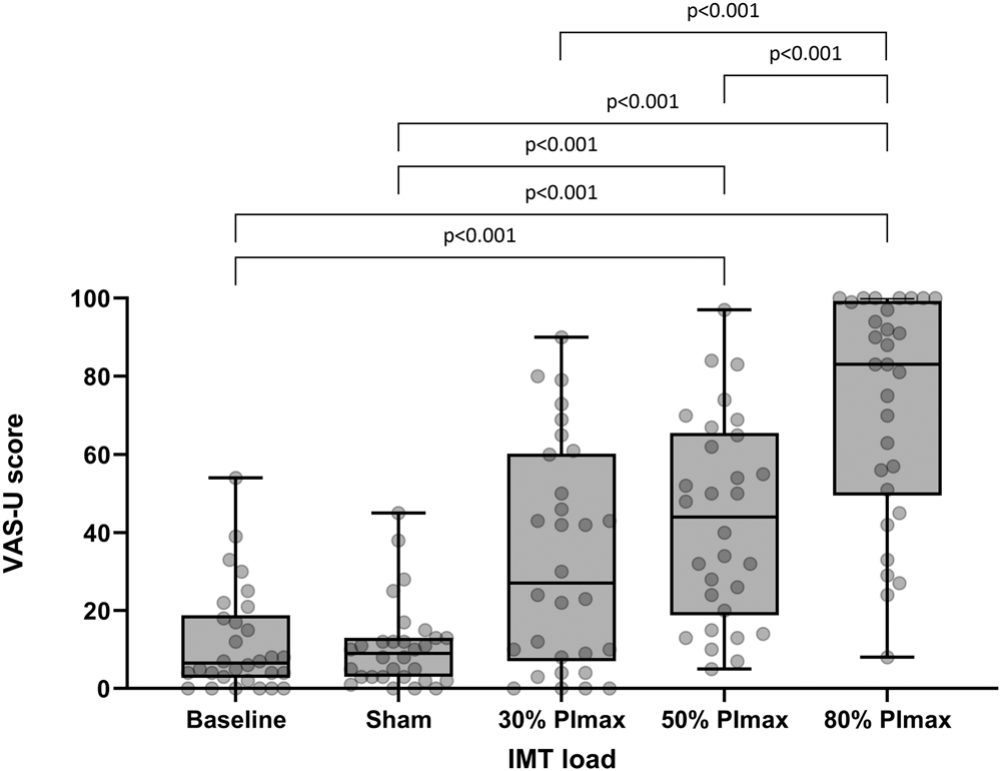
Title: Box and whisker plots detailing changes in visual analogue scale values for breathing unpleasantness (VAS‐U) at 0 (baseline), 4cmH_2_O (sham) and at 30, 50 and 80% IMT load. *p*‐values shown for significant relationships only. Individual data points are shown in background.

Median (IQR) VAS‐D and VAS‐U varied significantly with IMT dose (*p* < 0.001, Table 2, Figures 4 and 5). On *post hoc* testing, significant differences were seen between VAS‐D at baseline versus 30%, 50% and 80% (all <0.001), sham versus 30%, 50%, 80% (*p* = 0.048, *p* < 0.001, *p* < 0.001 respectively) and 30% and 50% versus 80% (*p* < 0.001, *p* = 0.048 respectively). Significant differences were seen between VAS‐U at baseline versus 50% and 80% of PImax (both *p* < 0.001), sham versus 50% and 80% of PImax (both *p* < 0.001), 30% versus 80% of PImax (*p* < 0.001), and 50% versus 80% of PImax (*p* = 0.022).

There were no significant differences in VAS‐U or VAS‐D between females and males at all IMT loads.

LMM showed a significant (*p* < 0.001) relationship between VAS‐D and load: intercept (95% CI) 9.20 (5.37–13.05) mm, slope (95%CI) 0.72 (0.61–0.83) mm/% PImax. Similar results were observed with VAS‐U: intercept (95% confidence interval) 8.80 (4.02–13.54) mm, slope (95%CI) 0.73 (0.61–0.84) mm/% PImax.

PImax significantly influenced the load‐perception relationship. Slope (95%CI) of load versus VAS‐D in the combined model 0.37 (0.09–0.65) mm/%PImax, *p* = 0.01), additional influence of baseline PImax 0.003 (0.001–0.005) mm/%PImax/cmH_2_O, *p* = 0.009, indicating that the load‐VAS‐D relationship became steeper by 0.003 mm/%PImax for each 1cmH_2_O increase in baseline strength.

## 7 DISCUSSION

We present data showing that oxygen consumption during inspiratory muscle training exhibits a significant positive dose relationship in the healthy respiratory system. There is also a significant positive relationship between VO_2_ and respiratory muscle effort relative to capacity. Perceived difficulty and unpleasantness of breathing increased with the applied threshold load. Underlying respiratory muscle strength exerts a significant additional influence on load perception.

### 7.1 Significance of findings

#### 7.1.1 Influence of IMT load on oxygen consumption

The load‐dependent increase in VO_2_ during IMT shown in our data is in agreement with other data in healthy individuals performing progressive inspiratory threshold loading (Collett et al., 1985; Eastwood et al., 1998), however, in one study, a fixed tidal volume was imposed (Collett et al., 1985). The changes observed were, while statistically significant, numerically small. Although numerically small, the changes are more pertinent if one considers that the increased oxygen consumption arose solely due to respiratory muscle contraction. Unique to our study, we measured VO_2_ at IMT loads commonly used in practice, whilst not imposing restrictions on tidal volume or respiratory rate, to replicate real‐world conditions; the data presented in this study supplements our previous work in mechanically ventilated patients, where we found a load‐dependent increase in VO_2_ during IMT (Jenkins et al., 2023). Whether the data from this study can be extrapolated to clinical populations is unclear; there are limited data drawing direct VO_2_ comparisons between healthy and diseased populations during loaded breathing. Baarends et al. found people with COPD have similar increases in VO_2_ during progressive loading compared to elderly adults (Baarends et al., 1998), with other work only testing oxygen consumption during hyperventilation in people with COPD, heart disease or obesity (Fritts et al., 1959; McGregor & Becklake, 1961).

#### 7.1.2 Perceived difficulty and unpleasantness of breathing during IMT

As expected, perceived difficulty and unpleasantness (measured by VAS‐D and ‐U) increased with the applied inspiratory load. To the authors' knowledge, this is the first study to measure perceived difficulty and unpleasantness during commonly cited IMT loads. Other authors have measured perceived difficulty, intensity, or effort during resisted breathing (Alexander‐Miller & Davenport, 2010; Knafelc & Davenport, 1999; Luu et al., 2022) (as a marker of perceived magnitude). However, these studies used the same respiratory load for all participants irrespective of PImax, making their findings less useful for IMT prescribers, where a percentage of PImax is commonly used. Our data clearly show a marked (and significant) increase in perceived difficulty and unpleasantness at 80% PImax compared to 50% PImax (median (IQR) 73 (48–93) mm and 79 (50–94) mm versus 41 (29–64) mm and 42 (20–55) mm, respectively) which should be taken into consideration when prescribing IMT, to help improve the experience of IMT, which, may in turn, help improve compliance with the intervention (Bissett et al., 2023).

Our data suggests that perceived difficulty is not only determined by the imposed load in relation to PImax, but also by the absolute magnitude of load. Presumably, the degree of corollary discharge is proportionate to the balance of load and capacity, but a higher absolute intrathoracic pressure during IMT may create more afferent feedback (perceived as greater breathing difficulty). This is a noteworthy observation when prescribing inspiratory muscle training interventions in practice; people with a higher inspiratory strength may find higher IMT loads more difficult than those with a lower inspiratory strength. Our observation requires replication and further exploration.

#### 7.1.3 Implications for IMT prescription

Our data indicate that IMT at 50% PImax may be optimal in terms of VO_2_ increase and tolerability; IMT at 50% PImax elicits a significant increase in VO_2_ from baseline (not present between baseline, sham or 30% PImax) (Figure 3), and avoids the marked increase in perceived difficulty and unpleasantness at 80% PImax compared to 50% PImax (Figures 4 and 5). The relationship between VAS‐D, VAS‐U and VO_2_ is detailed in Supplementary Figure 1, demonstrating a progressive separation of perceived difficulty and unpleasantness from VO_2_ as the IMT load increases. Whilst IMT at 50% PImax is frequently prescribed in studies investigating IMT (Bissett et al., 2023; Charususin et al., 2018b; Kilding et al., 2010; Romer et al., 2002; Turner et al., 2011), our data give those prescribing IMT further information on the physiological response, and the perceived difficulty and unpleasantness of this load, to better inform practice.

### 7.2 Strengths and limitations

This is the first study of its kind to measure oxygen consumption and perceived difficulty and unpleasantness during IMT. Our data provide valuable information on the relationship between load, perception and oxygen consumption, and open avenues for further investigation. Whilst our data indicate IMT at 50% PImax may be optimal for tolerability, factors other than load may improve adherence in clinical populations, such as self‐reported exertion to guide progression and automatic web‐based feedback (Sørensen & Svenningsen, 2018), or the use of electronic tapered flow resistive IMT, which can reduce perceived unpleasantness and breathing discomfort compared to threshold IMT (Langer et al., 2015; Van Hollebeke et al., 2023). Additionally, we only measured perceived difficulty and unpleasantness at a single time point; perception of IMT load may change after familiarization with IMT over an extended training period. We sought to replicate ‘real‐world’ conditions as best as possible, measuring the response to IMT using percentage PImax at commonly cited IMT loads to make the results more applicable to practice (Alexander‐Miller & Davenport, 2010; Beaumont et al., 2018; Charususin et al., 2018a; Elkins & Dentice, 2015; Knafelc & Davenport, 1999; Luu et al., 2022; Martin et al., 2011; Schultz et al., 2018). We did not control accessory respiratory muscle activation (McCool et al., 1989) or constrain tidal volume, breathing pattern or respiratory rate. Although Ti/Ttot did not influence the relationship between IMT load and VO_2_, we did not explore the influence of within‐ and between‐participant differences in respiratory rates or tidal volumes, which could have an impact on oxygen consumption (McCool et al., 1989). However, constraining the participants' breathing pattern and preventing them from making their normal, preferred breathing pattern changes during IMT would have impacted their perception of breathing difficulty and unpleasantness, and is not applicable to real‐world conditions. There was a non‐equal distribution of males and females in the recruited cohort;, however, the non‐significant differences in VO_2_, VAS‐U or VAS‐D between sex means this is unlikely to have influenced our results. For accurate measurement of VO_2_ the participant was required to wear a tight‐fitting mask throughout the testing period, which could increase perceived breathing difficulty and unpleasantness, perhaps explaining the non‐zero VAS‐D and VAS‐U scores at baseline.

## 8 CONCLUSIONS

This is the first study to examine the relationship between load, perception and VO_2_ during commonly cited IMT loads in healthy individuals. Our results show there is a significant relationship between IMT load and VO_2_. Perceived difficulty and unpleasantness increased with applied respiratory load. Underlying respiratory muscle strength exerts a significant relationship on load perception. These data indicate IMT at 50% PImax may be optimal in terms of VO_2_ increase and tolerability, providing additional information for those prescribing IMT, which may improve the experience and compliance of IMT in practice.

## AUTHOR CONTRIBUTIONS

**Timothy O. Jenkins**: Conceptualisation; methodology; validation; formal analysis; investigation; data curation; writing—original draft; writing—review & editing; project administration. **William Scott**: Data curation. **Christos Aristidou**: Data curation. **Mathias Krogh Poulsen**: Conceptualization; methodology; validation; writing—review & editing. **Stephen Edward Rees**: Conceptualization; methodology; validation; formal analysis; data curation; writing—review & editing. **Dan Stieper Karbing**: Conceptualization; methodology; validation; formal analysis; data curation; writing—review & editing. **Michael I. Polkey**: Conceptualization; methodology; validation; writing—review & editing; supervision. **Vicky MacBean**: Conceptualization; methodology; validation; formal analysis; writing—review & editing; supervision.

## CONFLICT OF INTEREST STATEMENT

The institution to which DSK and SER are affiliated has received funding from Mermaid Care A/S. DSK and SER have performed consultancy work for Mermaid Care A/S, which manufactured the Beacon Caresystem. SER was a board member and a minor shareholder in Mermaid Care A/S. MIP is a paid consultant for Philips Respironics; however, Philips had no role in this study.

## Supporting information


**Supplementary figure 1** The relationship between VAS‐D, VAS‐U and VO_2_. VAS‐D.

## Data Availability

Study data are avaliable at: https://doi.org/10.17633/rd.brunel.31021309.
