# Supplementary material for: Inspiratory muscle training in the healthy adult: The relationship between load, perception, and oxygen consumption
Source: Clin Physiol Funct Imaging. 2026 Jan 21;46(1):e70047. doi: 10.1111/cpf.70047 (PMC12820911; doi:10.1111/cpf.70047)
Supplement: Supplementary file 1 — Supplementary figure 1 The relationship between VAS‐D, VAS‐U and VO2. VAS‐D. [file CPF-46-0-s001.docx]

Supplementary file

**Supplementary figure 1**

The relationship between VAS-D, VAS-U and VO_2_. VAS-D: Green triangle; VAS-U: Blue Square; VO_2_: Red circle. Data shown as Mean (SD).
